# Current Understanding of Epidemiology, Pathophysiology, and Management of Atypical Femur Fractures

**DOI:** 10.1007/s11914-018-0464-6

**Published:** 2018-06-27

**Authors:** Jessica Starr, Yu Kwang Donovan Tay, Elizabeth Shane

**Affiliations:** 1 Division of Endocrinology, Department of Medicine, Columbia University Irving Medical Center, 180 Fort Washington Avenue, Room 9-910, New York, NY 10032 USA; 2 Department of Medicine, Sengkang General Hospital, Singapore, Singapore

**Keywords:** Atypical femur fracture, Bisphosphonates, Denosumab, Teriparatide, Hip geometry, Bone material properties

## Abstract

**Purpose of Review:**

To summarize reports published since the 2013 American Society of Bone and Mineral Research Task Force Report on atypical femoral fractures (AFF).

**Recent Findings:**

The absolute incidence of AFFs remains low. AFFs are primarily associated with prolonged bisphosphonate (BP) exposure, but have also been reported in unexposed patients and those receiving denosumab for osteoporosis and metastatic bone disease. Asians may be more susceptible to AFFs. Lateral femoral bowing and varus hip geometry, which increase loading forces on the lateral femoral cortex, may increase AFF risk. Altered bone material properties associated with BP therapy may predispose to AFFs by permitting initiation and increasing propagation of micro-cracks. Relevant genetic mutations have been reported in patients with AFFs. Single X-ray absorptiometry femur scans permit early detection of incomplete and/or asymptomatic AFFs. Orthopedists recommend intramedullary rods for complete AFFs and for incomplete, radiologically advanced AFFs associated with pain and/or marrow edema on MRI. Teriparatide may advance AFF healing but few data support its efficacy.

**Summary:**

Greater understanding of biological and genetic predisposition to AFF may allow characterization of individual risk prior to initiating osteoporosis therapy and help allay fear in those at low risk for this complication, which remains rare in comparison to the osteoporotic fractures prevented by antiresorptive therapy.

## Introduction

Low-energy femur fractures in patients receiving alendronate were first described in 2005 [1], followed by two case series in 2007 [2] and 2008 [3] reporting strong associations with alendronate. Since then, many articles have been published on atypical femur fractures (AFF). The American Society of Bone and Mineral Research Task Force on AFFs analyzed 310 published cases in 2010 [4]. In 2013, a second American Society for Bone and Mineral Research (ASBMR) Task Force Report on AFFs reviewed studies published between 2010 and 2013 [5]. It refined the case definition to emphasize the diagnostic importance of the periosteal stress reaction (beaking) at the site of fracture initiation in the lateral femoral cortex and the transverse orientation of the fracture line through the lateral cortex, summarized evidence that AFFs are stress fractures, and emphasized the importance of radiograph review versus hospital codes in epidemiological studies of AFFs [5]. The second report also summarized data on the absolute risk of AFFs in patients taking bisphosphonates (BPs), which ranged from 3.2 to 50 cases per 100,000 person-years for short-term use (< 5 years). However, long-term use (> 5 years) appeared to be associated with higher risk (~ 113 per 100,000 person-years). It summarized preliminary evidence that Asian race and lower limb geometry were risk factors for AFF [5]. In this article, we review papers published since 2013 that address the epidemiology, pathogenesis, and management of AFFs.

## Atypical Femur Fracture Case Definition

One goal of the ASBMR Task Force was to establish a case definition to distinguish AFFs from ordinary osteoporotic femoral shaft fractures. The AFF case definition published in the first ASBMR Task Force Report [4] and the revised case definition in the second ASBMR Task Force Report [5] differ in several respects (Table 1). The 2013 definition, which used newer data to improve the precision of the definition, made the location of the fracture (just below the lesser trochanter but above the supracondylar flare) a sine qua non, rather than a major feature, delineated five major features, and required that a minimum of four be present. As in the original definition, no minor features are required to be present. The major differences between the original and revised definitions are as follows: (1) periosteal or endosteal thickening of lateral cortex at the fracture site (“beaking or flaring”) was changed from a minor to a major feature, (2) minimal comminution was permitted, and (3) the wording “transverse or short oblique configuration” was expanded to specify that the fracture line must originate at the lateral cortex and remain transverse across the cortex, but permitted the fracture line to become oblique as it progressed medially across the femur. Recent studies, using the 2013 case definition and a variety of designs, report a low incidence of AFFs [6, 7••, 8••, 9, 10].

**Table 1:** Comparison of original and revised ASBMR case definition

	Original	Revised (changes from 2010 are in italicized font)
		*The fracture must be located along the femoral diaphysis from just distal to the lesser trochanter to just proximal to the supracondylar flare*
Major features	The fracture located anywhere along the femur from just distal to the lesser trochanter to just proximal to the supracondylar flare	
	Associated with no trauma or minimal trauma, as in a fall from a standing height or less	Associated with no trauma or minimal trauma, as in a fall from a standing height or less
	Transverse or short oblique configuration	*Fracture line originates at the lateral cortex and is substantially transverse in orientation*, *although it may become oblique as it progresses medially across the femur*
	Noncomminuted	Noncomminuted *or minimally comminuted*
	Complete fractures extend through both cortices and may be associated with a medial spike; incomplete fractures only involve lateral cortex	Complete fractures extend through both cortices and may be associated with a medial spike; incomplete fractures only involve lateral cortex
		Localized periosteal or endosteal thickening of lateral cortex at the fracture site (“beaking or flaring”)
Minor features	Localized periosteal reaction of lateral cortex (“beaking or flaring”)	
	Generalized increase in cortical thickness of the diaphysis	Generalized increase in cortical thickness of the *femoral diaphyses*
	Prodromal symptoms, such as dull or aching pain in groin or thigh	Unilateral or bilateral prodromal symptoms such as pain. B
	Bilateral fractures and symptoms	Bilateral incomplete or complete femoral diaphysis fractures
	Delayed healing	Delayed *fracture* healing

Several studies have addressed the effect of the new ASBMR criteria on the diagnosis of AFF. With regard to imaging techniques for diagnosis of AFFs, Critchlow et al. assessed the sensitivity and specificity of each radiographic criterion to identify an AFF [11]. Four independent experts representing different medical specialties within Kaiser Permanente Southern California compared radiographs from 55 AFFs and 39 non-AFFs. The most sensitive features distinguishing AFFs from non-AFFs were the lateral cortex transverse fracture pattern (mean 93.6%, range 85.5–98.2%), medial cortex transverse or oblique fracture pattern (mean 84.1%, range 72.7–98.2%), and minimal or non-comminution (mean 93.2%, range 89.1–98.2%). Specificity was greatest for lateral cortex transverse fracture pattern (mean 95.5%, range 92.3–97.4%). Luangkittikong and Unnanuntana reported similar prevalence of AFFs with both criteria and that localized periosteal thickening of the lateral cortex was the most specific finding for BP exposure in those with AFFs [12]. In a study by Orwoll and colleagues, two independent expert physicians applied the 2013 definition to radiographs previously categorized as AFFs by the 2010 definition [13]. The approximate 50% decrease in the number of fractures that met the 2013 than the 2010 ASBMR case definition (37 vs 74) was primarily due to the more precise specification of transverse configuration. Twelve shaft fractures were reclassified as AFFs, due to modification of comminution and periosteal/endosteal thickening criteria. In our opinion, radiographic studies that use the revised ASBMR case definition will capture the phenomenon more accurately.

On a cautionary note, Harborne et al. found that radiology reports are often inaccurate, with a high frequency of not reporting AFFs that meet ASBMR criteria and of improperly labeling fractures as AFFs when they do not meet criteria [14]. Inaccurate radiographic diagnosis of AFFs may adversely impact epidemiologic studies that attempt to characterize incidence and prevalence of AFF as well as patient management. In the latter case, if a fracture is improperly labeled an AFF, BPs or denosumab might be discontinued inappropriately. In contrast, not labeling a fracture that meets ASBMR criteria for an AFF may lead to inappropriate continuation of BPs or denosumab, which should be stopped upon diagnosis.

## Update on Epidemiology

In the second ASBMR Task Force report, AFF incidence was very low, ranging from 50 to 130 cases per 100,000 patient-years [5]. Their frequency was increased in patients on BPs, with a direct relationship between duration of BP exposure and risk of AFF [5, 15–23]. There was a significant association between glucocorticoid (GC) use and AFFs [5, 15, 18, 20, 22, 23]. Affected patients were approximately a decade younger than controls, a finding substantiated by a recent systematic review of 14 studies, in which 10 papers used the 2010 and 4 used the 2013 ASBMR definition [24••]. The overall incidence of AFFs was low ranging from 3.0 to 9.8 per 100,000 person-years [24••], the highest rate in a retrospective Norwegian fracture registry study that included periprosthetic fractures [9], which were specifically excluded in both ASBMR Task Force definitions. Other epidemiological studies have addressed relationships between AFF, BP use, and factors that may predispose certain patient populations to heightened risk. Most continue to report that AFF incidence is low, particularly compared to incidence of ordinary hip fractures [6–10, 25].

### Race

Confirming their earlier study suggesting Asian race as a risk factor for AFF [19, 26], Lo et al. analyzed diaphyseal femur fracture radiographs in women aged 50 and older who initiated oral BPs between 2002 and 2007 [7••]. The incidence of AFFs was eightfold higher in Asian than white women (64.2 vs 7.6 per 100,000 person-years) [7••]. Although exposed to BPs for slightly longer than white women (3.8 versus 2.7 years), the risk remained elevated for Asians (HR 6.6; 95% CI, 3.7–11.5) after adjustment for current and duration of BP use [7••]. In another prospective study with X-ray adjudication from New York, Marcano et al. found that AFFs were more likely to occur in Asians (OR 5.8; 95% CI, 1.69–19.62; *p* = 0.004) and patients of Hispanic descent (OR 5.8; 95% CI 1.43–23.22) [26].

Several studies addressed the incidence of and risk factors for AFFs in South Korea. A retrospective nested case-control study with radiograph review reported that AFFs were uncommon (0.12%), with an increased hazard ratio for long-term glucocorticoid use (3.0; 95% CI 1.403–6.568), prolonged BP exposure without drug holidays (5.2; 95% CI 2.0–13.362), and higher body mass index (1.2 per 1 kg/m^2^; 95% CI 1.109–1.371) [27]. Similarly, a prospective multicenter South Korean hip fracture study with radiograph adjudication that used the 2013 ASBMR case definition also found a low incidence of AFF (1.2%) that increased with BP exposure (OR 7.18, 95% CI 1.08–69.82, *p* = 0.045) and higher BMI (OR 1.17, 95% CI 1.13–1.88, *p* < 0.006) [28]. In a retrospective case-control study with radiograph adjudication that included only BP-treated patients, the age-adjusted incidence rate of AFFs was 72.7 per 100,000 person-years (95% CI, 29.1–175.8), with a sharp rise in incidence after more than 4 years of exposure [28, 29]. In conclusion, recent studies suggest women of Asian descent, particularly those taking glucocorticoids and those with higher BMIs, are at higher risk of AFF with BP exposure; however, Lee et al. found comparable incidence rates of AFF in BP-exposed Koreans and western European patients (Norway, Sweden, and Finland) [28, 29].

### Autoimmune Disease

Autoimmune disease and glucocorticoid use, established risk factors for osteoporotic fracture, have both been linked to AFF [10]. In 125 Japanese patients (90% women) with longstanding autoimmune disease taking BPs and glucocorticoids, Sato et al. reported that localized periosteal thickening of the lateral cortex (“beaking”) was present in 8.0% (15 femora, 10 patients) and new beaking developed in 10.3% (21 femora, 12 patients) over 2 years [30]. A complete AFF at the beaking site occurred in one patient. Factors significantly associated with beaking included > 4 years of BP therapy, longer duration of BP therapy (6.1 vs 5.0 years), age 40–60 years, and diabetes [30]. They measured the height of the beaking reaction in 20 femora (12 patients), characterizing it as pointed or arched [31]. Beaking was considered “severe” if associated with pain, a complete AFF, or an incomplete AFF with a visible fracture line; the periosteal reaction was higher and more commonly pointed in the severe form.

### AFFs in Patients with Osteoporosis Managed with Denosumab

AFFs have been reported in osteoporosis patients receiving denosumab. While the majority of reports document extensive prior BP exposure, as reviewed by Seiga et al. in 2016 [32] and reported by Ramchand et al. [33], AFFs have been reported in patients on denosumab with brief prior BP exposure [34]. In the FREEDOM Trial open-label extension, two participants developed AFFs (0.8 per 10,000 participant years), one after 7 years of denosumab exposure and one after 3 years of denosumab exposure [35••].

### AFFs in Patients with Osteoporosis Managed with Romosozumab

Romosozumab is a monoclonal antibody that increases bone formation by binding to and inhibiting sclerostin and also decreases bone resorption. In the Fracture Study of Postmenopausal Women with Osteoporosis (FRAME), one of 3521 participants in the romosozumab group had an AFF after 3.5 months of exposure; that individual had a history of prodromal pain at the fracture site prior to enrollment [36•]. In the Active-Controlled Fracture Study in Postmenopausal Women with Osteoporosis at High Risk (ARCH) study, 4093 postmenopausal women with osteoporosis and a fragility fracture were randomly assigned to monthly romosozumab or weekly oral alendronate for 12 months followed by open-label alendronate for another 12 months [37••]. There were no AFFs during in the initial 12 months in either group; in the second 12 months, two AFFs occurred in the romosozumab to alendronate group (< 0.1%) and four AFFs in the alendronate to alendronate group (0.2%).

### AFFs in Patients with Cancer Managed with Bisphosphonates and/or Denosumab

Edwards et al. retrospectively assessed the incidence of and risk factors for AFF in cancer patients followed at the MD Anderson Cancer Center over a 10-year period, both treated with oral and low-dose IV BPs for osteoporosis and with high-dose pamidronate and zoledronic acid for metastatic cancer [38]. As only AFFs that came to clinical attention were assessed, no absolute incidence rate was reported. Among 10,587 BP users, there were 23 AFFs, compared to two AFF cases among 300,553 patients who did not receive BPs (OR 355.58; 95% CI, 84.1 to 1501.4, *p* < 0.0001). In cancer patients treated for osteoporosis, six AFFs occurred in patients on alendronate for a mean of 84 months and two AFFs occurred in patients on ibandronate for a mean of 36 months. Compared to other BPs, the OR of an AFF was higher in patients treated with alendronate for osteoporosis (5.54; 95% CI; 1.60–19.112) and zoledronic acid was associated with a lower OR (0.34; 95% CI; 0.12–0.97). The authors hypothesized that the lower rate of AFFs in zoledronic acid users was because the drug concentrates in skeletal metastases and is less available to other skeletal sites [38]. However, there was a marked difference in duration of exposure between those treated with BPs for osteoporosis (84 and 36 months for alendronate and ibandronate, respectively) and those treated with zoledronic acid for metastatic cancer (5 and 14 months for zoledronic acid and pamidronate, respectively). Duration of exposure is an important risk factor for AFFs, as time is required for suppressed remodeling to cause changes in bone material properties (collagen and mineralization) that may predispose to micro-crack initiation and propagation [39].

Denosumab is used to treat metastatic skeletal disease and multiple myeloma at higher doses and with greater frequency than for osteoporosis (120 mg monthly vs 60 mg twice yearly). Tateiwa et al. reported two AFF patients with metastatic breast cancer; one took BPs for 11 years before starting denosumab and one took only BPs [40]. In both, tomosynthesis, an older three-dimensional imaging technique that permits acquisition of higher resolution images than conventional radiographs with lower radiation exposure than computed tomography, identified fracture lines within the area of cortical thickening that were not visible on radiographs [40]. Austin et al. reported two patients who sustained AFFs after receiving denosumab for metastatic cancer for two and 3.5 years without prior BP therapy [41•]. Both experienced prodromal thigh pain, and in both, the fractures were initially attributed to skeletal metastases; neither patient had histologic evidence of malignancy at the fracture site [41•]. Yang et al. reviewed records of 253 patients at their cancer center who received at least 12 doses of denosumab for metastatic bone disease. During a median follow-up of 27 months, they identified one patient with a complete AFF (incidence 0.4%; 95% CI 0.1–2.2%) who received 70 doses of IV BP before receiving 28 monthly doses of denosumab [42]. They also reviewed all available radiographs in a subset of 66 patients with at least 21 monthly doses of denosumab; two patients had diffuse cortical thickening of the femoral diaphysis and localized periosteal reaction of lateral femoral cortex (incidence 4.5%; 95% CI 1.6–12.5%), confirmed on bone scan and magnetic resonance imaging [42]. These papers raise concern that clinical and subclinical presentations of AFF may be attributed to metastases and missed in cancer patients.

### Periprosthetic AFFs

Two recent studies addressed periprosthetic fractures, which were excluded in the 2010 and the 2013 ASBMR Task Force case definitions because they are associated with a known risk of femoral fractures. A retrospective Norwegian study of all patients greater than or equal to 65 years old treated at a single institution between 2004 and 2011 for subtrochanteric and diaphyseal fractures included patients with and without implants [9]. Of 217 fracture patients with evaluable radiographs, 17 fractures in 16 women were designated atypical by unspecified criteria. Their catchment area included 21,630 women aged ≥ 65 years, of whom 2214 were treated with BPs. AFF incidence was 9.8 (95% CI 5.2 to 14.5) per 100,000 person-years and 79.0 (95% CI 37.8 to 120.3) per 100,000 person-years in those receiving BPs. However, 8 of 17 fractures occurred close to implanted metal [9]. A more recent 10-year retrospective study of 15 North American centers defined characteristics of 196 patients with AFFs receiving long-term (> 2 years) BPs in whom the AFF was periprosthetic (PAFF, *n* = 21) or not periprosthetic (AFF, *n* = 175) [43]. Only periprosthetic fractures with atypical features (lateral cortical beaking or hypertrophy, transverse lucency in the lateral cortex, transverse orientation of the fracture in the lateral cortex, minimal comminution) were included. PAFFs took longer to heal and had higher mortality and significantly more complications. Compared to the literature, several features common to patients with ordinary periprosthetic fractures (history of revision surgery, infection, total hip replacement for previous low-energy hip fracture with/without femoral loosening) were not present in BP-treated patients with PAFFs. Prodromal pain was common in PAFF patients but no data were presented [43]. While the ASBMR case definition for AFFs excluded periprosthetic fractures, emerging data suggest they may occur. Physicians should be alert to the radiographic and clinical features and consider immediate cessation of BP therapy, imaging of the contralateral limb, protected weight bearing, and close monitoring for signs of complete AFF or surgical fixation to stabilize the femur.

## Update on Pathogenesis

The second ASBMR Task Force considered AFFs to be stress or insufficiency fractures that develop over time, most commonly after prolonged suppression of bone remodeling by BPs, which may lead to osteon homogeneity with respect to tissue age and mineralization. In susceptible individuals, repetitive loading of the femur may lead to accumulation of micro-cracks within the cortex. Intracortical fracture repair, normally accomplished by targeted osteoclastic resorption of micro-cracks, may also be inhibited by BPs, which aggregate in actively remodeling bone, thus leading to micro-crack aggregation and propagation.

### Relationship Between Hip Geometry and AFFs

**Fig. 1:**
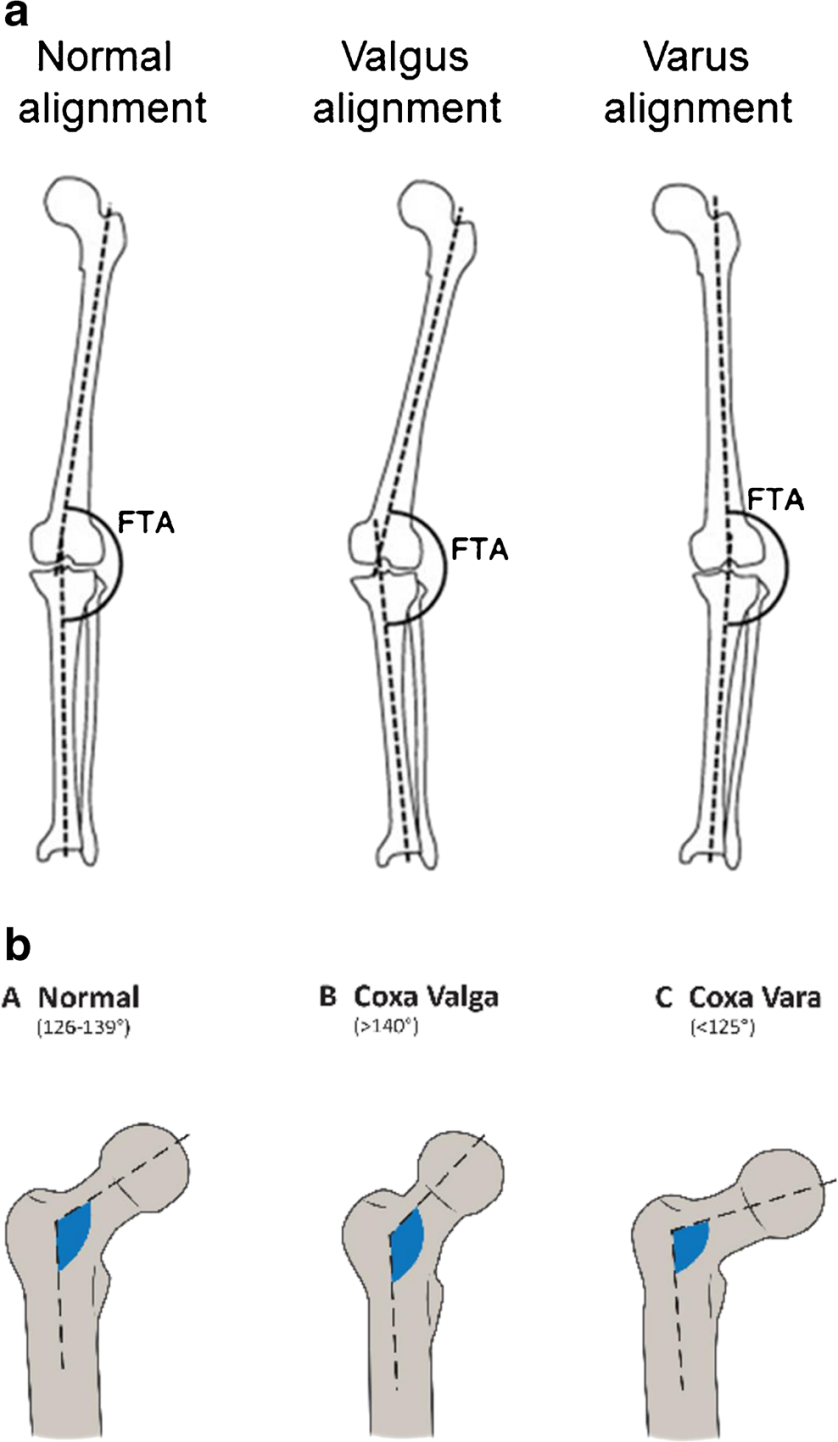

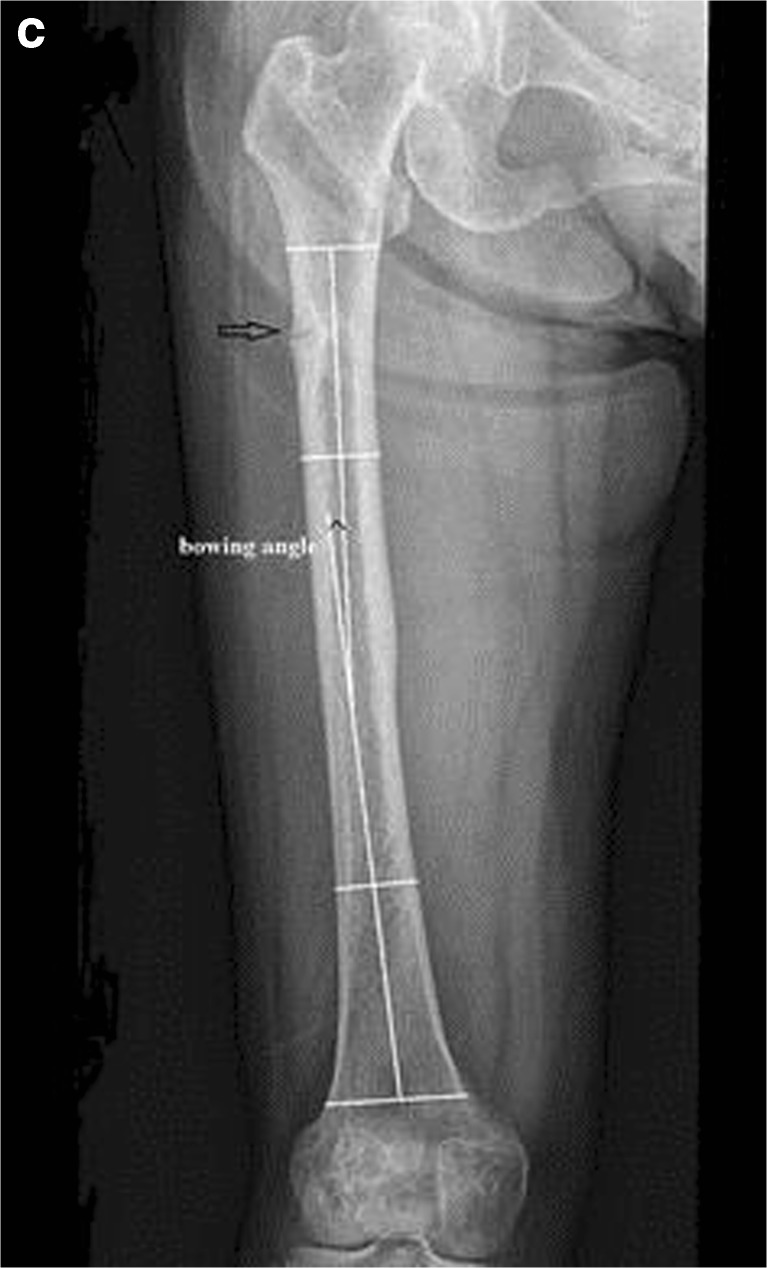
(**a**) Femorotibial angle: the femorotibial angle (FTA) is the lateral angle between the axis of the femoral shaft and that of the tibial shaft. An increased FTA is called varus alignment while a decreased FTA is called valgus alignment. (**b**) Femur neck-shaft angle: a decreased femur neck-shaft angle is called coxa vara or varus alignment. An increased neck-shaft angle is called coxa valga or valgus alignment. (**c**) Femoral bowing angle: femoral bowing angle is line that best describes the midpoint of the endosteal canal of the femoral diaphysis was drawn in the proximal and the distal quarters

The propensity for AFFs to be bilateral and in the same location on ipsilateral and contralateral sides suggests that hip geometry influences the position of maximal tensile stresses imposed on the lateral femoral cortex. Lateral femur bowing (Fig. 1a) and varus hip alignment (Fig. 1b) would increase tensile stress on the lateral femoral cortex, in turn increasing the risk of an AFF. Since the 2013 ASBMR Task Force Report, several publications support this concept. Saita et al. evaluated weight-bearing radiographs of 10 patients with 14 AFFs [44]. AFF locations were similar in those with bilateral fractures; the standing femorotibial angle (Fig. 1c) was significantly larger (more varus) in those with diaphyseal than subtrochanteric fractures and larger than those with ordinary femoral fractures [44]. In other studies, femoral neck-shaft angle was smaller in AFF patients than healthy controls in other studies, also suggesting that more varus proximal femoral geometry predisposes toward AFF [45–47]. A femoral neck-shaft angle cut-off of < 128.3° had a sensitivity of 69% and a specificity of 63% to predict AFF [47], although not observed in a Singaporean Chinese cohort [48••]. Chen et al. reported that diaphyseal AFFs tended to be associated with a larger lateral bowing angle whereas subtrochanteric AFFs tended to be associated with a smaller lateral bowing angle [49]. A study comparing Swedish and Asian Singaporean women found diaphyseal AFFs were common in Swedish and subtrochanteric AFFs in Singaporean women [21], in whom lateral femoral bowing was associated with more diaphyseal fracture locations [21]. A study using a 2D-3D X-ray scanner EOM™ technology showed that lateral femoral bowing angle was associated with AFF [50••]. The AFF location was influenced by both lateral femoral bowing angle and femoral neck-shaft angle, with subtrochanteric AFFs associated with more varus geometry and diaphyseal AFF with a smaller angle in a Caucasian population [50••]. A recent study of cadaveric femurs used patient-specific finite element (FE) modeling to quantify the relationship between femoral geometry and diaphyseal strain; small femur diameter and lateral and anterior bowing were associated with the highest femoral shaft strains [51]. In summary, there is increasing evidence that the presence of a more varus femorotibial angle and lateral femoral bowing influences mechanical forces on the lower limb and the region of maximal tensile loading on the lateral femoral cortex. Such biomechanical factors may account for the more proximal location of such fractures in individuals with more varus femorotibial angles. They may also predispose toward a higher rate of AFF by increasing the maximal tensile stresses imposed on the lateral femoral cortex.

### Genetic Predisposition

The first evidence for a genetic influence on AFFs was reported by Roca-Ayats et al. [52]. Whole exome sequencing in three sisters with AFFs and long-term BP therapy revealed a novel p.Asp188Tyr substitution in the enzyme geranylgeranyl pyrophosphate synthase Asp188Tyr located in the genomic position g.235505746G → T on chromosome 1 (GRCh37/hg19). This mutation in *GGPS1* affects a site within the enzyme that is inhibited by BPs, and this enzyme is key in the mevalonate pathway. This mutation would be expected to reduce enzyme activity and could predispose to AFF [52]. In a genome-wide search for non-synonymous variants in coding region between 13 AFF patients with and 286 controls without AFFs, 21 genetic variants were more common in the AFF group [53]. Many cases had two or more at risk variants suggesting that the risk for AFFs may be polygenic and result from accumulation of at risk genetic variants [53]. However, AFFs have been reported in BP-naïve patients, in patients using other anti-resorptives [32], and in other genetic conditions with suppressed bone turnover [54, 55] or defective mineralization [56, 57].

### Bone Material Properties in Patients with AFFs

Spontaneous or low-trauma fractures are unusual in the femur, which is rich in cortical bone and physiologically adapted to withstand large, repetitive forces. Although antiresorptive therapies increase bone mineral content, prolonged exposure may cause changes in cortical bone material properties with potentially deleterious effects on bone strength. The effects may vary by drug class. In a four-point bending study of femurs from osteoporotic sheep exposed to raloxifene, alendronate, zoledronate, or teriparatide for 1 year, alendronate was associated with reduced fatigue life (fewer cycles of stress before failure) and lower modulus loss at failure (reduced tendency for a material to bend) [58].

Biopsies of the proximal femoral cortex were compared among five groups of postmenopausal women undergoing surgery for fracture or total hip arthroplasty: BP-treated with AFF, BP-treated with ordinary osteoporotic fractures, BP-treated without fractures, BP-naïve with typical osteoporotic fractures, and BP-naïve without fractures [39]. By vibrational spectroscopy and nanoindentation, the BP-treated AFF group had higher tissue mineral content and more mature collagen (characteristics associated with bone that is harder and more brittle) than BP-treated women with ordinary osteoporotic fractures. In addition, BP-treated patients had increased propensity for crack initiation and decreased deflection of crack paths at osteon borders. This study showed that normal mechanisms by which bones dissipate energy and retard crack propagation were impaired by BPs; together with increased uniformity of mineralization, this could lower resistance to fracture and explain the transverse fracture morphology seen in AFFs.

Microindentation is a relatively new technology thought to provides an integrated measurement of the components of bone tissue (bone material properties) that contribute to bone material competence (strength or stiffness) at the nano- and micro level. The main outcome parameters, indentation distance increase (IDI), creep indentation distance (creep ID), and total indentation distance (total ID), capture the depth that an 11-Newton (N) force indents a standardized site at the midpoint of the anterior tibia. In general, higher measurements of IDI, creep ID, and total ID reflect poorer bone strength and decreased resistance to micro-crack propagation. Guerri-Fernandez et al. used in vivo microindentation to compare material properties of tibial bone among four groups of patients: BP-treated with AFF, BP-naïve with typical osteoporotic fractures, BP-treated without fractures, and BP-naïve without fractures (controls). After controlling for their older age, BP-treated AFF patients had higher total ID and IDI than controls (i.e., the 11-N force indented their tibias to a greater degree than the control group), suggesting their tibias were less resistant to crack propagation and thus not as strong [59]. The BP-naïve with typical osteoporotic fractures group had higher values than the BP non-users without fractures, suggesting that both patients with typical osteoporotic fractures and those with AFF have weak bone material properties [59].

In contrast, bone microarchitecture does not appear to influence AFF pathogenesis. Zanchetta et al. used high-resolution peripheral quantitative computed tomography (HR-pQCT) to evaluate microarchitecture among BP-treated AFF, BP-treated and BP-naïve patients without AFFs [60], finding no difference in any volumetric or microarchitectural index. However, as HR-pQCT measures bone microarchitecture at the radius and tibia, it could miss local changes in the femur.

### Mechanisms of Impaired Fracture Healing in AFF

Normally, bone micro-cracks heal by targeted remodeling in which osteoclasts resorb damaged tissue and osteoblasts form new bone. Suppression of remodeling, typical of BP-treated patients, has been documented in AFF patients by bone turnover markers, iliac crest biopsies, and fracture site biopsies [4, 5, 61]. Schilcher et al. performed micro-computed tomography (CT), infrared spectroscopy, and histomorphometry on cortical biopsies including the fracture line in eight patients, four with complete AFFs, and four with incomplete AFFs [62]. In the incomplete AFFs, the fracture gap varied from 150 to 200 μm wide and contained amorphous, nonmineralized, acellular necrotic material. Bone adjacent to the fracture gap demonstrated evidence of remodeling with osteoclasts, resorption cavities, and woven bone, with no evidence of remodeling or callus within the gap [62]. The investigators hypothesized that local strains related to low-impact activities such as walking prevented cell survival and delayed healing [62, 63]. Radiographic new bone deposition with bridging was observed within resected cortical deficits in all cases, within the expected time frame for cortical bone [64].

## Management

### Early Detection of AFFs

Between October 2011 and January 2013, 257 patients over age 50 who had been on BPs for over 5 years had a dual-energy X-ray absorptiometry (DXA) scan of the femur scan with the region of interest (ROI) extended distally from 15.3 to 22 cm. Cortical beaking was detected in 19 (7.4%); all had follow-up radiographs and seven (2.7%) had radiographic evidence of incomplete AFFs [65]. A subsequent study by the same investigators used single-energy (SE) DXA technology to image the entire femur between May 2013 and September 2014; none of 173 patients on BPs for over 5 years had cortical beaking, suggesting declining prevalence of AFFs possibly due to contemporaneous declines in BP prescribing from 2009 through 2014 [8••]. Between 2006 and 2014, Van de Laarschot et al. performed bilateral extended femur scans in 282 patients on long-term BPs [66]. Ten incomplete AFFs were diagnosed in nine patients (3.2%); one was a false positive and two patients did not have follow-up X-rays of the femur. Khosla et al., in a recent perspective in the *Journal of Bone and Mineral Research*, noted that SE DXA is a promising new technology that can detect localized periosteal reactions and may be useful to monitor patients who require long-term BPs for impending AFFs [67].

### Surgical Management

Internal fixation with intramedullary nailing is the mainstay of treatment for complete AFFs [4, 5, 68]. Prophylactic surgical intervention is also recommended for incomplete AFFs, particularly those with extensive cortical defects and pain and/or marrow edema on magnetic resonance imaging (MRI), which are predisposed to delayed or non-union or to progress to complete AFFs without surgical intervention [69, 70]. In a retrospective study of 11 patients with incomplete AFFs followed for an average of 10 months, one third became displaced, one third had persistent pain and/or progression of fracture line that necessitated surgery, and one third had persistent pain and no radiological evidence of healing [60]. Banffy et al. reported that five of six incomplete AFFs progressed to complete AFFs and that patients who underwent prophylactic surgery for incomplete AFF had shorter hospital stays than those who had surgery after a complete AFF [70]. Among complete AFF patients treated surgically, healing was slower than typical femur fracture and there was a high rate of non-union with 12% requiring revision surgery at an average of 11 months [71]. Patients with AFF shaft fractures were four times more likely to require reoperation than those with ordinary femoral shaft fracture; the most common reason for revision surgery was peri-implant fragility that would have been prevented with cephalomedullary nail [72]. In a recent survey of orthopedic surgeons, the preferred method to surgically fix complete or symptomatic incomplete AFF is with intramedullary nail [68]. Recently, Kharzami et al. reported good outcomes with a lateral plate in two patients with incomplete AFFs and significantly curved femurs [73••].

### Medical Management

Antiresorptive therapy should be discontinued immediately after diagnosis of a complete AFF [4, 5]. If periosteal thickening or cortical “beaking” of the lateral femoral cortex is detected, it is critical to evaluate the patient for cortical lucency with magnetic resonance imaging (MRI) or computed tomography (CT). MRI (and bone scintigraphy) also detects bone or marrow edema/hyperemia in addition to the cortical lucency. In one study, BP withdrawal was associated with a reduced risk of AFF by 70% every year [21]. In another, six of nine fractures healed after stopping alendronate [1]. In a retrospective review of 12 patients on long-term glucocorticoid therapy and BPs, continuing BPs for 2 years after detecting cortical “beaking” was associated with radiological progression [31].

Although teriparatide is commonly prescribed to accelerate fracture healing in AFFs, evidence to support its efficacy remains weak. In a small uncontrolled study of 14 patients with AFF treated with teriparatide for 24 months, spine BMD improved by 6.1% and bone turnover markers increased significantly; of 4 patients with incomplete fractures who did not undergo surgery, 1 healed, 1 partially healed, and 2 showed no healing [61]. A retrospective review of 16 complete AFFs treated with intramedullary rods found a trend for shorter time to union and significantly better 6-month functional outcomes in the eight who received teriparatide [74]. The Fix-IT study randomized 13 women with an AFF to 12 months of teriparatide immediately versus after 6 months [75]. There was a trend for superior radiographic healing at 6 and 12 months in the immediate vs delayed group. Because the number of patients was small, the results must be interpreted with caution. However, since a randomized controlled trial of sufficient size is unlikely to be conducted, it may be reasonable to use teriparatide as the potential benefits may outweigh any risks.

In general, when teriparatide is used to treat osteoporosis, it must be followed by antiresorptive therapy in order to maintain gains in bone mass that occurred on therapy. In our opinion, whether teriparatide therapy in AFF patients must be followed by antiresorptive therapy depends on the indication for which teriparatide is prescribed. If a relatively brief course of teriparatide is prescribed to accelerate AFF healing, the patient might not require subsequent antiresorptive therapy. However, if teriparatide is prescribed because the patient remains severely osteoporotic and it is felt necessary to increase bone mass and decrease risk of future osteoporotic fractures, then discontinuation after 24 months will result in loss of the newly formed bone and declines in BMD; antiresorptive therapy would be necessary to prevent this well-known phenomenon. However, re-exposing an AFF patient to antiresorptive therapy after teriparatide may precipitate recurrence of AFFs. In this regard, Ramchand et al. reported a patient with bilateral incomplete AFFs that healed after a year of teriparatide and recurred after 6 months of denosumab [33]. In our opinion, great caution is warranted when considering reinstitution of antiresorptive therapy in an AFF patient with incomplete AFFs. The risk of recurrence might be lower in a patient who had had a complete AFF that was managed surgically with an intramedullary rod. However, in the setting of a unilateral, surgically managed AFF, the risk to the contralateral limb should be considered.

## Conclusions

This paper summarizes recent key findings on the epidemiology, pathogenesis, and management of AFF since the 2013 ASBMR Task Force recommendations were issued. We believe that the revised case definition has led to greater specificity in the diagnosis of AFFs, but under- and inaccurate reporting of AFFs remain a problem. Our understanding of the epidemiology of AFFs remains essentially unchanged: low absolute incidence rates that may be declining in recent years possibly because of decreased BP prescribing, strong associations with BP therapy particularly of long duration, more evidence for an association with Asian race, and emerging evidence for an association with prosthetic implants. There is also increasing evidence that hip and lower limb geometry, genetic predisposition, and changes in bone material properties influence their pathogenesis. Recent development of single-energy DXA scan technology that can detect incipient cortical “beaking” may permit monitoring of patients on long-term antiresorptive therapy for incomplete AFFs prior to fracture. This could alleviate physician and patient concern about AFFs and improve rates of treatment initiation and compliance. Greater understanding of the biological and genetic pathogenesis of AFF may permit a more precise approach to assessing individual risk before starting antiresorptive therapy. This may allay fears of this complication, which remains rare in comparison to the osteoporotic fractures prevented by antiresorptive therapy. Lastly, strong evidence for improved fracture healing of AFF with teriparatide is limited, but teriparatide may be of some benefit in accelerating AFF fracture healing.

## References

[1] Odvina CV, Zerwekh JE, Rao DS, Maalouf N, Gottschalk FA, Pak CYC (2005). Severely suppressed bone turnover: a potential complication of alendronate therapy. J Clin Endocrinol Metab.

[2] Goh SK, Yang KY, Koh JS, Wong MK, Chua SY, Chua DT (2007). Subtrochanteric insufficiency fractures in patients on alendronate therapy: a caution. J Bone Joint Surg Br.

[3] Neviaser AS, Lane JM, Lenart BA, Edobor-Osula F, Lorich DG (2008). Low-energy femoral shaft fractures associated with alendronate use. J Orthop Trauma.

[4] Shane E, Burr D, Ebeling PR, Abrahamsen B, Adler RA, Brown TD, et al. Atypical subtrochanteric and diaphyseal femoral fractures: report of a task force of the American Society for Bone and Mineral Research. J Bone Miner Res. 2010;25(11):2267–94. 10.1002/jbmr.253 20842676

[5] Shane E, Burr D, Abrahamsen B, Adler RA, Brown TD, Cheung AM, et al. Atypical subtrochanteric and diaphyseal femoral fractures: second report of a task force of the American Society for Bone and Mineral Research. J Bone Miner Res. 2014;29(1):1–23. 10.1002/jbmr.1998 23712442

[6] Juby AG, Crowther S, Cree M (2014). Identifying atypical femoral fractures—a retrospective review. Calcif Tissue Int.

[7] •• Lo JC, Hui RL, Grimsrud CD, Chandra M, Neugebauer RS, Gonzalez JR, et al. The association of race/ethnicity and risk of atypical femur fracture among older women receiving oral bisphosphonate therapy. Bone. 2016;85:142–7. **This paper compared incidence rates of AFFs in > 48,000 women who initiated BPs and found an incidence rate of 18.7 per 100,000 person-years overall that was eightfold higher in Asian compared to white women even after adjusting for age and treatment duration.** 10.1016/j.bone.2016.01.002 PMC5108728 26769007

[8] •• McKenna MJ, McKiernan FE, McGowan B, Silke C, Bennett K, van der Kamp S, et al. Identifying incomplete atypical femoral fractures with single-energy absorptiometry: declining prevalence. J Endocr Soc. 2017;1(3):211–20. **This paper demonstrates the ability of single energy to visualize incomplete AFFs.** 10.1210/js.2016-1118 PMC5686782 29264478

[9] Meling T, Nawab A, Harboe K, Fosse L (2014). Atypical femoral fractures in elderly women: a fracture registry-based cohort study. Bone Joint J.

[10] Takakubo Y, Ohta D, Ishi M, Ito J, Oki H, Naganuma Y, et al. The incidence of atypical femoral fractures in patients with rheumatic disease: Yamagata Prefectural Committee of Atypical Femoral Fractures (YamaCAFe) Study. Tohoku J Exp Med. 2017;242(4):327–34. 10.1620/tjem.242.327 28883214

[11] Adams AL, Xue F, Chantra JQ, Dell RM, Ott SM, Silverman S, et al. Sensitivity and specificity of radiographic characteristics in atypical femoral fractures. Osteoporos Int. 2017;28(1):413–7. 10.1007/s00198-016-3809-y 27766369

[12] Luangkittikong S, Unnanuntana A (2014). Prevalence of atypical femoral fractures in Thai patients at a single institution. J Med Assoc Thail.

[13] LeBlanc ES, Rosales AG, Black DM, Genant HK, Dell RM, Friess DM (2017). Evaluating atypical features of femur fractures: how change in radiological criteria influenced incidence and demography of atypical femur fractures in a community setting. J Bone Miner Res.

[14] Harborne K, Hazlehurst JM, Shanmugaratnam H, Pearson S, Doyle A, Gittoes NJ, et al. Compliance with established guidelines for the radiological reporting of atypical femoral fractures. Br J Radiol. 2016;89(1057):20150443. 10.1259/bjr.20150443 PMC4985957 26508355

[15] Girgis CM, Sher D, Seibel MJ (2010). Atypical femoral fractures and bisphosphonate use. N Engl J Med.

[16] Giusti A, Hamdy NA, Dekkers OM, Ramautar SR, Dijkstra S, Papapoulos SE (2011). Atypical fractures and bisphosphonate therapy: a cohort study of patients with femoral fracture with radiographic adjudication of fracture site and features. Bone.

[17] Lenart BA, Neviaser AS, Lyman S, Chang CC, Edobor-Osula F, Steele B, et al. Association of low-energy femoral fractures with prolonged bisphosphonate use: a case control study. Osteoporos Int. 2009;20(8):1353–62. 10.1007/s00198-008-0805-x PMC4415520 19066707

[18] Feldstein AC, Black D, Perrin N, Rosales AG, Friess D, Boardman D, et al. Incidence and demography of femur fractures with and without atypical features. J Bone Miner Res. 2012;27(5):977–86. 10.1002/jbmr.1550 22275107

[19] Lo JC, Huang SY, Lee GA, Khandewal S, Provus J, Ettinger B, et al. Clinical correlates of atypical femoral fracture. Bone. 2012;51(1):181–4. 10.1016/j.bone.2012.02.632 22414379

[20] Meier RPH, Perneger TV, Stern R, Rizzoli R, Peter RE (2012). Increasing occurrence of atypical femoral fractures associated with bisphosphonate use. Arch Intern Med.

[21] Schilcher J, Michaelsson K, Aspenberg P (2011). Bisphosphonate use and atypical fractures of the femoral shaft. N Engl J Med.

[22] Thompson RN, Phillips JR, McCauley SH, Elliott JR, Moran CG (2012). Atypical femoral fractures and bisphosphonate treatment: experience in two large United Kingdom teaching hospitals. J Bone Joint Surg Br.

[23] Dell RM, Adams AL, Greene DF, Funahashi TT, Silverman SL, Eisemon EO, et al. Incidence of atypical nontraumatic diaphyseal fractures of the femur. J Bone Miner Res. 2012;27(12):2544–50. 10.1002/jbmr.1719 22836783

[24] Khow KS, Shibu P, Yu SC, Chehade MJ, Visvanathan R (2017). Epidemiology and postoperative outcomes of atypical femoral fractures in older adults: a systematic review. J Nutr Health Aging.

[25] Donnelly KJ, Tucker A, Kerr B, McDonald S, O’Longain DS, Acton JD (2017). A review of atypical subtrochanteric femoral fractures in Northern Ireland between 2010 and 2014. Eur J Orthop Surg Traumatol.

[26] Marcano A, Taormina D, Egol KA, Peck V, Tejwani NC (2014). Are race and sex associated with the occurrence of atypical femoral fractures?. Clin Orthop Relat Res.

[27] Koh JH, Myong JP, Yoo J, Lim YW, Lee J, Kwok SK, et al. Predisposing factors associated with atypical femur fracture among postmenopausal Korean women receiving bisphosphonate therapy: 8 years’ experience in a single center. Osteoporos Int. 2017;28(11):3251–9. 10.1007/s00198-017-4169-y 28748389

[28] Lee YK, Kim TY, Ha YC, Song SH, Kim JW, Shon HC, et al. Atypical subtrochanteric fractures in Korean hip fracture study. Osteoporos Int. 2017;28(10):2853–8. 10.1007/s00198-017-4112-2 28612307

[29] Lee YK, Ahn S, Kim KM, Suh CS, Koo KH (2018). Incidence rate of atypical femoral fracture after bisphosphonates treatment in Korea. J Korean Med Sci.

[30] Sato H, Kondo N, Wada Y, Nakatsue T, Iguchi S, Fujisawa J, et al. The cumulative incidence of and risk factors for latent beaking in patients with autoimmune diseases taking long-term glucocorticoids and bisphosphonates. Osteoporos Int. 2016;27(3):1217–25. 10.1007/s00198-015-3382-9 26519417

[31] Sato H, Kondo N, Nakatsue T, Wada Y, Fujisawa J, Kazama JJ, et al. High and pointed type of femoral localized reaction frequently extends to complete and incomplete atypical femoral fracture in patients with autoimmune diseases on long-term glucocorticoids and bisphosphonates. Osteoporos Int. 2017;28(8):2367–76. 10.1007/s00198-017-4038-8 28409215

[32] Selga J, Nunez JH, Minguell J, Lalanza M, Garrido M (2016). Simultaneous bilateral atypical femoral fracture in a patient receiving denosumab: case report and literature review. Osteoporos Int.

[33] Ramchand SK, Chiang CY, Zebaze RM, Seeman E (2016). Recurrence of bilateral atypical femoral fractures associated with the sequential use of teriparatide and denosumab: a case report. Osteoporos Int.

[34] Khow KS, Yong TY (2015). Atypical femoral fracture in a patient treated with denosumab. J Bone Miner Metab.

[35] •• Bone HG, Wagman RB, Brandi ML, Brown JP, Chapurlat R, Cummings SR, et al. 10 years of denosumab treatment in postmenopausal women with osteoporosis: results from the phase 3 randomised FREEDOM trial and open-label extension. Lancet Diabetes Endocrinol. 2017;5(7):513–23. **This paper describes the FREEDOM Trial open-label extension and reports that two participants developed AFFs, one during year 7 and one during year 3 of denosumab. The incidence rate was 0.8 per 10,000 participant years or 8.0 per 100,000 participant years, comparable to incidence rates in bisphosphonate-treated patients.** 10.1016/S2213-8587(17)30138-9 28546097

[36] • Cosman F, Crittenden DB, Adachi JD, Binkley N, Czerwinski E, Ferrari S, et al. Romosozumab treatment in postmenopausal women with osteoporosis. N Engl J Med. 2016;375(16):1532–43. **This paper reported rates of AFFs in cancer patients followed over 10 years and treated with low-dose BPs for osteoporosis or high dose BPs for metastatic cancer. The odds ratio was much higher in BP-treated than non-BP-treated patients and higher in patients treated for osteoporosis with alendronate than zoledronic acid. The duration of BP exposure was higher in those treated for osteoporosis than for cancer.** 10.1056/NEJMoa1607948 27641143

[37] •• Saag KG, Petersen J, Brandi ML, Karaplis AC, Lorentzon M, Thomas T, et al. Romosozumab or alendronate for fracture prevention in women with osteoporosis. N Engl J Med. 2017;377(15):1417–27. **Using spectroscopic imaging, this biopsy study demonstrated that normal mechanisms by which bones dissipate energy and retard crack propagation were impaired by BPs; together with increased uniformity of mineralization, this could lower resistance to fracture and explain the transverse fracture morphology seen in AFFs.** 10.1056/NEJMoa1708322 28892457

[38] Edwards BJ, Sun M, West DP, Guindani M, Lin YH, Lu H, et al. Incidence of atypical femur fractures in cancer patients: the MD Anderson Cancer Center experience. J Bone Miner Res. 2016;31(8):1569–76. 10.1002/jbmr.2818 26896384

[39] Lloyd AA, Gludovatz B, Riedel C, Luengo EA, Saiyed R, Marty E, et al. Atypical fracture with long-term bisphosphonate therapy is associated with altered cortical composition and reduced fracture resistance. Proc Natl Acad Sci. 2017;114(33):8722–7. 10.1073/pnas.1704460114 PMC5565436 28760963

[40] Tateiwa D, Outani H, Iwasa S, Imura Y, Tanaka T, Oshima K, et al. Atypical femoral fracture associated with bone-modifying agent for bone metastasis of breast cancer: a report of two cases. J Orthop Surg (Hong Kong). 2017;25(3):2309499017727916. 10.1177/2309499017727916 28844196

[41] Austin DC, Torchia MT, Klare CM, Cantu RV (2017). Atypical femoral fractures mimicking metastatic lesions in 2 patients taking denosumab. Acta Orthop.

[42] Yang SP, Kim TW, Boland PJ, Farooki A (2017). Retrospective review of atypical femoral fracture in metastatic bone disease patients receiving denosumab therapy. Oncologist.

[43] Robinson Jde D, Leighton RK, Trask K, Bogdan Y, Tornetta P (2016). Periprosthetic atypical femoral fractures in patients on long-term bisphosphonates: a multicenter retrospective review. J Orthop Trauma.

[44] Saita Y, Ishijima M, Mogami A, Kubota M, Baba T, Kaketa T, et al. The fracture sites of atypical femoral fractures are associated with the weight-bearing lower limb alignment. Bone. 2014;66:105–10. 10.1016/j.bone.2014.06.008 24933347

[45] Mahjoub Z, Jean S, Leclerc J-T, Brown JP, Boulet D, Pelet S, et al. Incidence and characteristics of atypical femoral fractures: clinical and geometrical data. J Bone Miner Res. 2016;31(4):767–76. 10.1002/jbmr.2748 26588590

[46] Hagen JE, Miller AN, Ott SM, Gardner M, Morshed S, Jeray K, et al. Association of atypical femoral fractures with bisphosphonate use by patients with varus hip geometry. JBJS. 2014;96(22):1905–9. 10.2106/JBJS.N.00075 25410509

[47] Taormina DP, Marcano AI, Karia R, Egol KA, Tejwani NC (2014). Symptomatic atypical femoral fractures are related to underlying hip geometry. Bone.

[48] •• Chou ACC, Ng ACM, Png MA, Chua DTC, Ng DCE, Howe TS, et al. Bone cross-sectional geometry is not associated with atypical femoral fractures in Asian female chronic bisphosphonate users. Bone. 2015;79(Supplement C):170–5. **This study found that AFF location was influenced by lateral femoral bowing angle and femoral neck-shaft angle, with subtrochanteric AFFs associated with more varus geometry and diaphyseal AFF with a smaller angle in a Caucasian population.** 10.1016/j.bone.2015.06.003 26067179

[49] Chen L-P, Chang T-K, Huang T-Y, Kwok T-G, Lu Y-C (2014). The correlation between lateral bowing angle of the femur and the location of atypical femur fractures. Calcif Tissue Int.

[50] •• Morin SN, Wall M, Belzile EL, Godbout B, Moser TP, Michou L, et al. Assessment of femur geometrical parameters using EOS™ imaging technology in patients with atypical femur fractures; preliminary results. Bone. 2016;83:184–9. **This study provided first evidence for a genetic influence on AFFs in three sisters with AFFs and long-term BP therapy who had a novel mutation in the gene encoding the enzyme geranylgeranyl pyrophosphate synthase, a site inhibited by BPs in the mevalonate pathway. This mutation would be expected to reduce enzyme activity and could predispose to AFF.** 10.1016/j.bone.2015.10.016 26541215

[51] Haider IT, Schneider P, Michalski A, Edwards WB (2018). Influence of geometry on proximal femoral shaft strains: implications for atypical femoral fracture. Bone.

[52] Roca-Ayats N, Balcells S, Garcia-Giralt N, Falcó-Mascaró M, Martínez-Gil N, Abril JF, et al. GGPS1 mutation and atypical femoral fractures with bisphosphonates. N Engl J Med. 2017;376(18):1794–5. 10.1056/NEJMc1612804 28467865

[53] Pérez-Núñez I, Pérez-Castrillón JL, Zarrabeitia MT, García-Ibarbia C, Martínez-Calvo L, Olmos JM, et al. Exon array analysis reveals genetic heterogeneity in atypical femoral fractures. A pilot study. Mol Cell Biochem. 2015;409(1):45–50. 10.1007/s11010-015-2510-3 26160281

[54] Yates CJ, Bartlett MJ, Ebeling PR (2011). An atypical subtrochanteric femoral fracture from pycnodysostosis: a lesson from nature. J Bone Miner Res.

[55] Birmingham P, McHale KA. Case reports: treatment of subtrochanteric and ipsilateral femoral neck fractures in an adult with osteopetrosis. Clin Orthop Relat Res. 2008;466(8):2002–8. 10.1007/s11999-008-0256-x PMC2584256 18431613

[56] Whyte MP (2009). Atypical femoral fractures, bisphosphonates, and adult hypophosphatasia. J Bone Miner Res.

[57] Sutton RA, Mumm S, Coburn SP, Ericson KL, Whyte MP (2012). “Atypical femoral fractures” during bisphosphonate exposure in adult hypophosphatasia. J Bone Miner Res.

[58] Brock GR, Chen JT, Ingraffea AR, MacLeay J, Pluhar GE, Boskey AL, et al. The effect of osteoporosis treatments on fatigue properties of cortical bone tissue. Bone Rep. 2015;2:8–13. 10.1016/j.bonr.2014.10.004 PMC4306187 25642445

[59] Guerri-Fernandez RC, Nogues X, Quesada Gomez JM, Torres Del Pliego E, Puig L, Garcia-Giralt N (2013). Microindentation for in vivo measurement of bone tissue material properties in atypical femoral fracture patients and controls. J Bone Miner Res.

[60] Zanchetta MB, Diehl M, Buttazzoni M, Galich A, Silveira F, Bogado CE, et al. Assessment of bone microarchitecture in postmenopausal women on long-term bisphosphonate therapy with atypical fractures of the femur. J Bone Miner Res. 2014;29(4):999–1004. 10.1002/jbmr.2107 24115250

[61] Watts NB, Aggers D, McCarthy EF, Savage T, Martinez S, Patterson R (2017). Responses to treatment with teriparatide in patients with atypical femur fractures previously treated with bisphosphonates. J Bone Miner Res.

[62] Schilcher J, Sandberg O, Isaksson H, Aspenberg P (2014). Histology of 8 atypical femoral fractures. Acta Orthop.

[63] Gustafsson A, Schilcher J, Grassi L, Aspenberg P, Isaksson H (2016). Strains caused by daily loading might be responsible for delayed healing of an incomplete atypical femoral fracture. Bone.

[64] Bögl HP, Aspenberg P, Schilcher J (2017). Undisturbed local bone formation capacity in patients with atypical femoral fractures: a case series. Osteoporos Int.

[65] McKenna MJ, van der Kamp S, Heffernan E, Hurson C (2013). Incomplete atypical femoral fractures: assessing the diagnostic utility of DXA by extending femur length. J Clin Densitom.

[66] van de Laarschot DM, Smits AA, Buitendijk SK, Stegenga MT, Zillikens MC (2017). Screening for atypical femur fractures using extended femur scans by DXA. J Bone Miner Res.

[67] Khosla S, Cauley JA, Compston J, Kiel DP, Rosen C, Saag KG, et al. Addressing the crisis in the treatment of osteoporosis: a path forward. J Bone Miner Res. 2016;32:424–30. 10.1002/jbmr.3074. 10.1002/jbmr.3074 28099754

[68] Schneider PS, Wall M, Brown JP, Cheung AM, Harvey EJ, Morin SN (2017). Atypical femur fractures: a survey of current practices in orthopedic surgery. Osteoporos Int.

[69] Ha Y-C, Cho M-R, Park KH, Kim S-Y, Koo K-H (2010). Is surgery necessary for femoral insufficiency fractures after long-term bisphosphonate therapy?. Clin Orthop Relat Res.

[70] Banffy MB, Vrahas MS, Ready JE, Abraham JA (2011). Nonoperative versus prophylactic treatment of bisphosphonate-associated femoral stress fractures. Clin Orthop Relat Res.

[71] Bogdan Y, Tornetta PI, Einhorn TA, Guy P, Leveille L, Robinson J (2016). Healing time and complications in operatively treated atypical femur fractures associated with bisphosphonate use: a multicenter retrospective cohort. J Orthop Trauma.

[72] Schilcher J (2015). High revision rate but good healing capacity of atypical femoral fractures. A comparison with common shaft fractures. Injury.

[73] Kharazmi M, Michaelsson K, Hallberg P, Schilcher J (2018). Lateral fixation: an alternative surgical approach in the prevention of complete atypical femoral fractures. Eur J Orthop Surg Traumatol.

[74] Yeh WL, Su CY, Chang CW, Chen CH, Fu TS, Chen LH, et al. Surgical outcome of atypical subtrochanteric and femoral fracture related to bisphosphonates use in osteoporotic patients with or without teriparatide treatment. BMC Musculoskelet Disord. 2017;18(1):527. 10.1186/s12891-017-1878-5 PMC5729282 29237448

[75] Greenspan SL, Vujevich K, Britton C, Herradura A, Gruen G, Tarkin I, et al. Teriparatide for treatment of patients with bisphosphonate-associated atypical fracture of the femur. Osteoporos Int. 2018;29(2):501–6. 10.1007/s00198-017-4286-7 PMC6468986 29085957

